# Discrimination of Clover and Citrus Honeys from Egypt According to Floral Type Using Easily Assessable Physicochemical Parameters and Discriminant Analysis: An External Validation of the Chemometric Approach

**DOI:** 10.3390/foods7050070

**Published:** 2018-05-03

**Authors:** Ioannis K. Karabagias, Sofia Karabournioti

**Affiliations:** 1 Laboratory of Food Chemistry, Department of Chemistry University of Ioannina, 45110 Ioannina, Greece; 2 Attiki Bee Culturing Co., Alex. Pittas S.A, Protomagias 9, Kryoneri 14568 Athens, Greece; skar@attiki-pittas.gr

**Keywords:** Egyptian honey, physicochemical parameters, characterization, discrimination, chemometrics, quality control

## Abstract

Twenty-two honey samples, namely clover and citrus honeys, were collected from the greater Cairo area during the harvesting year 2014–2015. The main purpose of the present study was to characterize the aforementioned honey types and to investigate whether the use of easily assessable physicochemical parameters, including color attributes in combination with chemometrics, could differentiate honey floral origin. Parameters taken into account were: pH, electrical conductivity, ash, free acidity, lactonic acidity, total acidity, moisture content, total sugars (degrees Brix-°Bx), total dissolved solids and their ratio to total acidity, salinity, CIELAB color parameters, along with browning index values. Results showed that all honey samples analyzed met the European quality standards set for honey and had variations in the aforementioned physicochemical parameters depending on floral origin. Application of linear discriminant analysis showed that eight physicochemical parameters, including color, could classify Egyptian honeys according to floral origin (*p* < 0.05). Correct classification rate was 95.5% using the original method and 90.9% using the cross validation method. The discriminatory ability of the developed model was further validated using unknown honey samples. The overall correct classification rate was not affected. Specific physicochemical parameter analysis in combination with chemometrics has the potential to enhance the differences in floral honeys produced in a given geographical zone.

## 1. Introduction

Honey was an important staple in Ancient Egypt, a civilization that was relatively advanced in the fields of mathematics, medicine, architecture, and astronomy. Many wall paintings portray honey from the period described or make references to honey. The Ebers Papyrus, a medical document written 1550 years before Christ, has been reported to contain 147 recipes that enhance honey. Ancient Egyptians used honey as both a sweetener and a medicinal remedy. Egypt, Spain, and Greece have been beekeeping the longest. Currently, the number of honeybee colonies in Egypt is larger than that in both Spain and Greece. The total annual production of honey in Egypt is estimated to be 110 tons. Clover, thyme, citrus, and cotton honeys are the most pronounced honey types that contribute to the total honey production in Egypt [1].

There are indicative research studies that have been carried out over the past 40 years, in different parts of the world, focusing on the characterization and authentication of honey using melissopalynological, conventional, or instrumental methods in combination with chemometrics [2,3,4,5,6,7,8,9,10,11,12,13,14,15].

Authentication of honey may be termed as the accurate determination of its botanical and geographical origin with respect to its unique composition and properties.

Melissopalynological analysis is the official method for the botanical origin determination of honey [16]. 

However, as the world goes forward, people tend to look for simple, economical, and accurate methods of analysis, especially in the case of foodstuffs. Conventional methods of analysis are widely recognized and may serve as helpful tools for the characterization of honey in contrast to the more complicated instrumental methods of analysis, which require the use of cost effective solvents, standard compounds, trained analysts, etc. The same holds for the melissopalynological analysis, which is in general characterized as a difficult procedure for the floral origin determination of honey [17]. 

Yet, there is no study that attempts to characterize and differentiate the floral type of domestic Egyptian honeys using easily assessed physicochemical parameters in combination with supervised statistical methods, in terms of quality control and authentication. 

Thus, the aim of the present study was to characterize and, if possible, differentiate Egyptian clover and citrus honeys according to floral origin using a set of simple and reproducible tests for physicochemical parameters in combination with supervised statistical tools.

## 2. Materials and Methods 

### 2.1. Honey Samples

Twenty-two Egyptian honeys, namely clover (15 samples, *Trifolium alexandrinum* L.) and citrus (7 samples, *Citrus* sp.), were collected from the greater Cairo area during the harvesting year 2014–2015. Honey samples from Egypt were packaged in glass containers, shipped to the laboratory, and maintained at 4 ± 1 °C until analysis, which was accomplished at the earliest opportunity. For the external validation of the developed discrimination model, six honey samples collected from mountainous parts of Arkadia (Peloponnese, Greece) were used and served as the ‘’unknown’’ samples. 

### 2.2. Melissopalynological Analysis

The floral origin of Egyptian honeys was confirmed by the melissopalynological analysis [16]. 

### 2.3. Reagents and Solutions

Sodium hydroxide, hydrochloric acid (37%), ethanol pro analysis, and phenolphthalein used for the determination of free and lactonic acidity were purchased from Sigma Aldrich (Germany). 

### 2.4. Determination of Conventional Physicochemical Parameters

The physicochemical parameters pH, electrical conductivity (EC), free acidity (FA), lactonic acidity (LA), and total acidity (TA) (the sum of free and lactonic acidity) were determined according to International Honey Commission methods [18]. The lactonic/free acidity ratio (L/FA) was calculated by dividing lactonic acidity by free acidity values. Ash content was determined on the basis of electrical conductivity results [18]. Moisture and total sugar content (°Bx) were determined using a hand held refractometer (ATC, Bellingham + Stanley, UK). Results reported are the average values of three determinations (*n* = 3).

### 2.5. Determination of Salinity and Total Dissolved Solids (TDS)

Salinity and total dissolved solids of a 20% (*w*/*v*) honey solution in distilled water were measured at 20 °C using a Delta OHM, model HD 3456.2, conductimeter (Padova, Italy) coupled with 4-ring and 2-ring conductivity/temperature probes. Temperature was measured by 4 wire Pt 100 and 2 wire Pt 1000 sensors by immersion. The probe was calibrated automatically, using a conductivity standard solution (1413 μS/cm) which was purchased from Hannah (Hannah Instruments, Inc., Woonsocket, RI, USA). Results were expressed as g/L and mg/L, respectively. Finally, total dissolved solids/total acidity ratio (TDS/TA) was calculated by dividing total dissolved solids by total acidity values. All measurements were performed in triplicate (*n* = 3).

### 2.6. Determination of Color Parameters (L*, a*, b*) and Browning Index 

The surface color of honeys considering three parameters L*, a*, b* of the CIELAB (CIE -Commission Internationale de l’ Eclairage) system was measured according to Karabagias et al. [11]. In particular, color parameter L* corresponds to degree of brightness, parameter a* (positive values) corresponds to degree of redness, a* (negative values) corresponds to degree of greenness, parameter b* corresponds to yellowness of color (when positive) and to blueness of color (when negative) [19]. Browning index was determined using color chromaticity coordinates (see Section 3.1.3). The results reported are the average values of five determinations (*n* = 5).

### 2.7. Statistical Analysis

Multivariate analysis of variance (MANOVA) and linear discriminant analysis (LDA) were applied to the investigated set of data and computed by the statistics software SPPS (version.20.0 for windows). MANOVA was applied to all the investigated physicochemical and color parameters, as a pre-treatment procedure, in order to point out the significant parameters that could differentiate Egyptian honeys according to floral origin (*p* < 0.05). 

LDA, which is a supervised statistical technique, was then applied only to the significant parameters (*p* < 0.05) (independent variables) to determine a linear combination of these group of subjects, which could provide a discrimination rate of Egyptian honeys according to floral origin (clover and citrus) (dependent variables). The original and cross validation methods were considered. In the cross validation method, each case is classified by the functions derived from all cases other than that case. Finally, the statistical criterion of Wilk’s Lambda was also considered, since it evaluates the statistical significance (discriminatory power) of the discriminant functions derived [13].

## 3. Results and Discussion

### 3.1. Melissopalynological Analysis

In most cases, a honey is considered as coming predominantly from a given botanical origin (unifloral honey) if the relative frequency of the pollen of that *taxon* exceeds 45%. Therefore, the following terms are used in order to characterize the relative frequency of the main pollen types: predominant pollen >45%, secondary pollen 16–45%, important minor pollen 3–15%, and minor pollen <3% [16].

During the microscopic examination of honeys, variations in characteristic pollen of clover and citrus honeys were monitored (Table 1). Based on the frequency of pollen grains encountered, the botanical origin of clover honey samples was in accordance with package labeling (average value of predominant pollen *Trifolium alexandrinum* was ca. 69%). The average number of citrus pollen grains was ca. 32%, covering the group of secondary pollen. However, there were cases where citrus pollen grains were under-represented in numerous European citrus honeys (range of pollen grain percentages between 2–42%) [16]. In that sense, melissopalynological data may be combined with those of physicochemical or sensory data for the accurate characterization of a monofloral honey [16]. Therefore, Egyptian honeys analyzed in the present study were categorized as clover and citrus based on the melissopalynological and physicochemical parameter analyses carried out. 

#### 3.1.1. Physicochemical Parameter Values of Egyptian Honeys

Data regarding physicochemical and color parameters of Egyptian honeys are summarized in Table 2. Clover and citrus honeys recorded significant variations in TDS, salinity, EC, moisture, ash, moisture, FA, TA, TDS/TA, and ash values. These honey types belong to blossom honeys, hence, physicochemical parameter analysis showed significant differences on the aforementioned parameters (*p* < 0.05). However, pH, total sugar content, browning index (BI), LA, and L/FA did not vary significantly according to floral origin of honeys (Table 3).

Free acidity, moisture content, along with electrical conductivity values conform to the European directive relating to blossom honey [20]. What is remarkable is the fact that clover and citrus honeys showed very low acidity values, indicating possible regional characteristics

Moisture content of Egyptian clover honeys is in agreement with that of Argentinean [21] and Pakistanean [22] clover honeys and within the range reported for Algerian blossom honeys [9]. However, clover honey from India possessed higher moisture content [23].

FA and TA values of Egyptian clover honeys are significantly lower than those reported in the aforementioned studies [9,21,22,23]. However, LA values of Egyptian clover honeys are in excellent conformity with those of India [23].

At this point, it should be noted that there is no data in the literature involving L/FA ratio for Egyptian clover honeys. EC values of Egyptian clover honeys revealed that they are a typical blossom honey [20]. 

pH values are in agreement with those of clover honey from Pakistan [22]. Finally, ash content is much lower compared to Pakistanean and Indian clover honeys [22,23]. 

Egyptian citrus honeys recorded lower FA values than citrus honeys from Morocco [4,17], Pakistan [22], and Greece [11]. However, lactonic acidity was much higher compared to citrus honeys from Pakistan [22] or Greece [11] and within the range of that reported for Moroccan citrus honeys [22]. L/FA was lower than that of Moroccan citrus honeys [12] and higher of that reported for Greek citrus honeys [11]. Furthermore, EC and ash values of Egyptian citrus honeys are lower compared to those of Greek citrus honeys [11]. Finally, pH values are within the range reported for Moroccan [4,12] and Greek citrus honeys [11].

#### 3.1.2. Salinity and TDS Values of Egyptian Honeys

Salinity may be termed as the saltiness or amount of salt dissolved in a body of water (solution). Total dissolved solids (TDS) are a measure of the combined content of all inorganic and organic substances contained in a liquid, in molecular, ionized or micro-granular (colloidal sol) suspended form. In the present study, salinity and TDS served as tools for the floral differentiation of Egyptian honeys, since significant variations were recorded among the different floral types (Table 2). The lower TDS and salt content was recorded for citrus honeys. In a study involving Algerian honeys [8] TDS were significantly higher than those of the present study. However, it should be noted that this is the first report on the salt and TDS content for Egyptian honeys and there are very limited articles in the literature upon the determination/and or use of these physicochemical parameters. 

#### 3.1.3. Color and Browning Index Values of Egyptian Honeys

Honey color is the primary criterion of quality, acceptance, and preference among different types of consumers. It varies from light to almost black amber tones, with the most common being bright yellow, orange, or reddish. Parameters that may affect honey color are: (i) botanical origin; (ii) storage time; (iii) flavonoid content; (iv) ash content; (v) the temperature of honey at the hive, (vi) the use of new or already used (old) hives for honey collection, etc., [24].

Egyptian citrus honeys analyzed were the brightest honeys (had the higher mean L* values compared to the mean values of clover or thyme honeys) (Table 2). The results regarding the L* color parameter were in line with those of Petretto et al. [15] involving Moroccan citrus honeys. Color parameter a* had negative values (green components) for all the analyzed honeys (Table 2). The a* values were within the range reported previously for citrus honeys produced in Greece [11], but significantly lower than those reported for native Mexican honeys [10].

Additionally, color parameter b* (yellow components) had positive and rather constant values for all honeys analyzed. Present b* values for clover and citrus honeys from Egypt are higher than those reported for Moroccan blossom honeys [5] and within the range reported for Greek citrus honeys [11]. 

Browning index (BI) is a measure of the development of brown color in foodstuffs. Recently, Tornuk et al. [25] reported that the brown pigment development in honey was associated with thermal processing due to the non-enzymatic browning such as the Maillard reaction. At the same time, the Maillard reaction depends, to a great extent, on the presence of a high concentration of sugar and amino acids under thermal conditions. Based on the aforementioned, a browning reaction could occur as a consequence of increased temperature during honey processing. However, it is well known that browning may also arise from prolonged honey storage at room temperature. 

Present results showed that all honeys were not thermally treated, since browning index values were much lower as compared to thermally or ultrasound treated commercial honeys [26]. This comprises an additionally quality criterion for Egyptian clover and citrus honeys for the domestic or international markets. It should be stressed that this is the first report in the literature regarding BI values of clover and citrus honeys produced in Egypt. Finally, browning index determination could serve as: (i) a fast qualitative criterion of honey thermal treatment investigation, since it is free and simpler to carry out, compared to HMF or diastase number determination and (ii) the determination of honey storage time at room temperature.

#### 3.1.4. Discrimination of Egyptian Honey According to Floral Type Based on Selected Physicochemical Parameter Values 

Eight selected significant physicochemical parameters values, namely L*, a*, TDS, salinity, moisture, FA, TA, and TDS/TA (Table 3) were subjected to linear discriminant analysis. Results showed that one discriminant function was formed: Wilks’ Lambda = 0.204, X^2^ = 25.418, df = 8, *p* < 0.01. The discriminant function 1 was used for the classification of Egyptian honeys according to floral origin, since it explained 100% of total variance providing an eigenvalue of 3.897 and a good canonical correlation equal to 0.892. In addition, the standardized canonical discriminant function coefficients for each of the significant physicochemical parameters that contributed to the floral discrimination of Egyptian honeys are given in Table 4. The overall correct classification rate was 95.5% for the original and 90.9% for the cross validation method, considered a satisfactory discrimination rate for this method. The higher discrimination rate was provided for clover (93.3%) followed by citrus (85.7%) honeys. 

Discrimination ability of conventional physicochemical parameters, ease of application, and reproducibility, have been previously reported in the literature in studies involving Spanish [3,6,13], Moroccan [12], and Greek [11] unifloral honeys, in agreement with the present results. What is remarkable, is that the discrimination rate obtained for citrus honeys is in great agreement with the results (classification rate of 82%) reported by Terrab et al. [4] involving native Moroccan honeys (*Citrus* sp., *Lythrum* sp. and Apiaceae). Serrano et al. [6] classified Spanish eucalyptus and citrus honeys using electrical conductivity and water activity values in combination with linear discriminant analysis. The overall correct classification rate, based on the cross validation method, was higher than that of the present study (96.6%). However, the number of the investigated parameters (i.e., botanical origin, physicochemical parameters, etc.) may affect the overall correct classification rate. 

#### 3.1.5. Summary Regarding the Identification of the Variables with the Highest Discriminatory Power 

The higher the absolute value of a standardized canonical coefficient, the more significant the variable is for the determination of honey origin [13]. Table 3 shows the standardized canonical discriminant function coefficients obtained in the developed statistical model for the discrimination of clover and citrus honeys from Egypt. Based on the aforementioned, the variables that most contributed to the discrimination of Egyptian honeys according to floral origin were color parameter a*, TDS, TA, and the ratio of TDS to TA. What is worth mentioning is that the ratio of total dissolved solids to total acidity (TDS/TA) recorded significant differences among clover and citrus honeys and so may be proposed as a new index of honey botanical origin. Research on a larger number of honey samples belonging to different honey types will confirm further the present hypothesis. 

#### 3.1.6. External Validation of the Developed Statistical Model for the Differentiation of Egyptian Honeys According to Floral Type

In order to investigate the robustness of the statistical model developed for the classification of clover and citrus honeys from Egypt, unpublished data involving specific physicochemical parameters of honeys from Greece were introduced into the set of data and a new statistical analysis was carried out. Honeys from Greece served as the ‘’unknown’’ honey samples. The common physicochemical parameter values taken into account from our database were moisture, free acidity, total sugars (°Bx), browning index, pH, electrical conductivity, CIE color (L*, a*, b*), TDS, and salinity. Thus, these physicochemical parameters served as the independent variables while botanical origin (clover, citrus, and unknown honeys) was taken as the dependent variable. The total number of honey samples was increased to 28 prior to discriminant analysis. Based on CIE color parameter analysis, visual color estimation, electrical conductivity, and ash content values, the unknown honey samples from Greece could be classified as honeydew honeys (Table 5).

**Figure 1:**
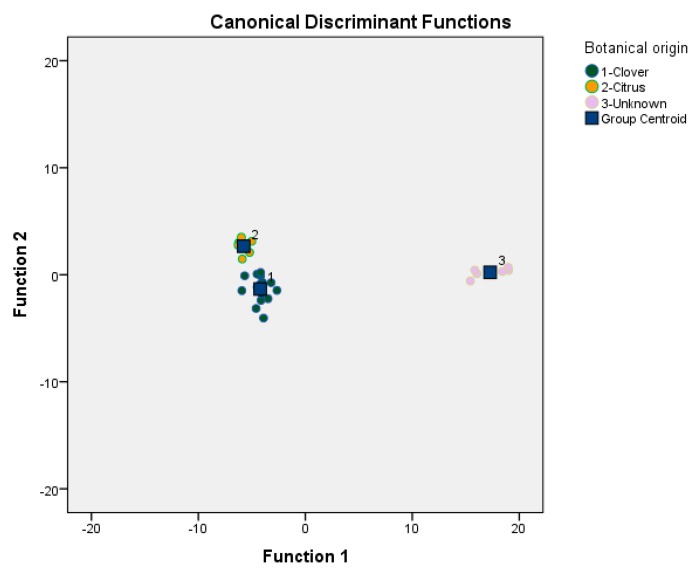
Floral discrimination of clover and citrus honeys from Egypt against ‘’unknown’’ honeys from Greece based on 11 physicochemical parameters.

Discriminant analysis showed that two discriminant functions were formed: Wilks’ Lambda = 0.003, X^2^ = 121.611, df = 20, *p* < 0.001 for the first and Wilks’ Lambda = 0.246, X^2^ = 28.774, df = 9, *p* = 0.001 for the second. However, discriminant function 1 was the basic function for the classification of Egyptian and unknown honeys according to floral origin, since it explained 96.8% of the total variance providing a high eigenvalue (91.630) and a high canonical correlation (0.995) in comparison with those of the discriminant function 2 (eigenvalue of 3.070 and canonical correlation of 0.869). Respective group centroid values, representing the discriminant functions created, were (−4.220, −1.334), (−5.766, 2.666), and (17.277, 0.224), for clover, citrus, and unknown honeys, respectively (Figure 1).

In Figure 1 it is also shown that clover, citrus, and unknown honeys are well differentiated. The overall correct classification rate was 100% for the original and 92.9% for the cross validation method, which is considered a very satisfactory discrimination rate for this method. The higher discrimination rate was provided for citrus (100%) followed by clover (93.3%), and unknown (83.3%) honeys. Table 6 lists the discriminatory power of the developed statistical model.

### 3.2. Formatting of Mathematical Components

Browning index (BI) could be estimated as follows, BI = $\left[100\times \left(\frac{x-0.31}{0.172}\right)\right]$ where *x* = $\frac{a*+1.75L*}{5.645L*+a*-3.012b*}$, was estimated according to Ferrari et al. [27] using L*, a*, and b* color coordinates.

## 4. Conclusions

Results of the present study showed that specific conventional physicochemical and color parameters in combination with MANOVA and LDA may differentiate the floral origin of Egyptian honeys even when produced in domestic/and or close distance zones, in agreement with previous works in the literature [3,4,5,6,11,13,21,28]. Physicochemical parameters may be easily assessed in routine honey quality control, since these are, in general, less complicated than other methods, for example, instrumental methods of analysis. Authentication of honey is a matter of great interest worldwide, since it involves and protects a wider audience, including producers, exporters, and consumers, from adulterated or false labeled, processed, impure, or from unknown origin honeys that may enter the global market.

## Figures and Tables

**Table 1:** Full pollen characteristics (% pollen grains) of clover and citrus honeys from Egypt.

Clover Honeys (N = 15)	*Trifolium alexandrinum*	*Melilotus* sp.	*Brassica* sp.	*Helianthus annuus*	*Umbelliferae*	*Eucalyptus* sp.	Compositae	Labiatea	Nectarless:	Minor Pollen
1	41%	22%	16%	6%	6%	-	3%	-	*Zea mays*	*Ononis* sp. 2%
2	56%	3%	26%	6%	3%	3%	-	-	*Zea mays, Gramineae*	<1% *Sesamum* sp., *Musa* sp*. Pheonix* sp, *Punica* sp.
3	67%	-	13%	6%	6%	-	3%	-	*Zea mays, Gramineae*	<1% *Musa* sp*, Pheonix* sp.
4	68%	9%	7%	-	9%	-	3%	-	*Zea mays, Gramineae*	<1% *Musa* sp, *Pheonix* sp, *Eucalyptus* sp.
5	79%	2%	-	-	9%		3%	-	*Zea mays, Gramineae*	*Vicia* sp. 1%, *Phoenix* sp.1%, <1% *Eucalyptus* sp., Labiatea
6	51%		-	-	1%	45%	2%		*Zea mays*	<1% *Eucalyptus* sp., *Pheonix* sp.
7	89%		-	-	1%	5%	1%	-	*Zea mays*	<1% *Citrus sp.*, *Musa sp.*
8	91%	2%	-	-	-	2%	-	1%	*Zea mays, Gramineae*	
9	56%		-	-	3%	40%		-	*Zea mays, Gramineae*	*Vicia* sp. 1%
10	76%	1%	-	-	1%	12%	6%	-	*Gramineae*	*Vicia* sp. 1%
11	73%	2%	-	-	7%	8%	-	8%	*Zea mays, Gramineae*	<1% *Musa* sp., *Sesamum* sp., Compositae
12	63%	3%	-	-	5%	18%	-	6%	*Zea mays*	*Vicia* sp. 1%, <1% *Musa* sp., *Sesamum* sp., Gossypium
13	79%	-	-	-	1%	18%	-	-	*Zea mays*	<1% *Musa* sp., *Sesamum* sp. *Vicia* sp., *Melilotus* sp., Labiatea
14	68%	-	1%	-	1%	27%	-	-	*Zea mays*	<1% *Musa* sp., *Sesamum* sp. *Vicia* sp., *Melilotus,* Labiatea
15	70%	-	6%	-	7%	12%	-	-	*Zea mays*	*Vicia* sp. 2%, <1% *Musa* sp., *Sesamum* sp, *Melilotus,* Labiatea
Citrus honeys (N = 7)	*Umbelliferae*	*Citrus* sp.	*Eucalyptus* sp.	*Trifolium alexandrinum*	*Pheonix* sp.	*Brassica* sp.	Compositae	Labiatea	Nectarless	
1	36%	28%	22%	8%	3%	-	-	-	*Gramineae*	<1% *Casuaria* sp.*, Ephorbia* sp*.*
2	*36%*	36%	24%	1%	1%	12%	-	-	*Gramineae*	
3	29%	18%	14%	18%	<1%		3%		*Zea mays*	<1% Compositae, *Brassica* sp., *Diplotaxis* sp.
4	32%	38%	-	6%	-	3%	18%	-	*Zea mays, Gramineae*	
5	12%	42%	28%	3%	1%	1%	8%	-	*Zea mays, Gramineae*	<1% *Sesamum* sp., *Diplotaxis* sp.
6	8%	32%	-	3%	1%	1%	6%	-	*Zea mays, Gramineae*	-
7	33%	32%	22%	1%	1%	1%	6%	-	*Zea mays, Gramineae*	-

N: number of honey samples.

**Table 2:** Physicochemical parameters of clover and citrus honeys from Egypt.

Botanical Origin	L*	a*	b*	TDS (mg/L)	Salinity (g/L)	pH	EC (mS/cm)	Ash (g/100g)	Moisture (g/100g)	FA (meq/kg)	LA (meq/kg)	TA (meq/kg)	L/FA	BI	TDS/TA	Total Sugars (°Bx)
Clover	76.14	−2.65	7.56	112.2	0.108	3.38	0.224	0.048	15.50	6.50	7.35	13.85	1.13	7.53	8.10	82.60
Clover	75.88	−2.85	7.76	126.2	0.120	3.61	0.252	0.064	15.95	8.00	6.05	14.05	0.76	7.64	8.98	82.55
Clover	76.05	−3.07	8.34	129.4	0.124	3.49	0.259	0.068	15.55	6.50	5.95	12.45	0.92	8.22	10.39	82.85
Clover	76.72	−2.79	6.93	132.6	0.126	3.46	0.265	0.072	16.25	8.00	6.15	14.15	0.77	6.46	9.37	82.20
Clover	75.7	−3.06	9.42	131.0	0.124	3.4	0.263	0.071	15.50	7.00	5.85	12.85	0.84	9.82	10.19	83.00
Clover	76.69	−3.65	5.88	127.2	0.120	3.31	0.254	0.066	17.05	7.50	5.90	13.40	0.79	4.20	9.49	81.10
Clover	76.45	−3.9	6.98	117.6	0.112	3.27	0.235	0.055	16.75	8.00	5.75	13.75	0.72	5.48	8.55	81.60
Clover	75.88	−3.14	4.83	114.7	0.109	3.26	0.228	0.051	16.80	7.00	5.95	12.95	0.85	3.32	8.86	81.55
Clover	74.28	−2.55	8.90	146.0	0.140	3.78	0.298	0.091	17.45	9.50	6.45	15.95	0.68	9.77	9.15	81.00
Clover	76.45	−2.26	5.40	137.7	0.127	3.5	0.275	0.078	15.55	7.00	5.75	12.75	0.82	4.91	10.80	80.75
Clover	76.68	−2.29	5.40	138.4	0.129	3.41	0.276	0.078	18.00	7.50	6.95	14.45	0.93	4.86	9.58	80.20
Clover	73.82	−2.61	10.96	148.6	0.140	3.48	0.296	0.090	17.35	8.50	4.65	13.15	0.55	12.87	11.30	81.10
Clover	74.15	−2.71	9.33	158.2	0.150	3.51	0.314	0.100	17.25	9.00	6.10	15.10	0.68	10.26	10.48	81.10
Clover	75.13	−2.85	8.51	157.4	0.144	3.50	0.307	0.096	17.35	11.00	6.00	17.00	0.55	8.79	9.26	81.00
Clover	73.23	−2.23	10.22	150.5	0.142	3.46	0.300	0.092	17.85	9.00	7.05	16.05	0.78	12.24	9.38	80.55
Average	75.55	−2.84	7.76	135.2	0.13	3.45	0.270	0.075	16.68	8.00	6.13	14.13	0.78	7.76	9.59	81.54
±SD	1.15	0.48	1.86	14.71	0.01	0.13	0.029	0.017	0.89	1.24	0.64	1.36	0.15	2.90	0.88	0.89
Citrus	74.98	−4.5	11.63	134.2	0.128	3.61	0.267	0.073	17.45	8.50	5.85	14.35	0.69	11.75	9.35	80.95
Citrus	76.1	−4.85	11.78	132.6	0.127	3.48	0.253	0.065	17.55	8.50	6.70	15.20	0.79	11.44	8.72	81.55
Citrus	76.84	−3.23	4.37	95.20	0.091	3.4	0.193	0.030	18.00	6.50	6.20	12.70	0.95	2.58	7.50	82.05
Citrus	77.49	−3.4	4.00	90.10	0.088	3.39	0.187	0.027	17.35	7.00	5.55	12.55	0.79	1.92	7.18	82.15
Citrus	77.79	−3.56	4.18	82.10	0.078	3.43	0.164	0.014	17.25	5.00	6.15	11.15	1.23	1.99	7.36	81.80
Citrus	76.9	−3.22	4.36	81.20	0.078	3.31	0.159	0.011	17.35	5.50	5.35	10.85	0.97	2.58	7.48	81.95
Citrus	77.7	−3.36	3.90	94.00	0.090	3.36	0.185	0.026	17.85	6.50	5.20	11.70	0.80	1.82	8.03	82.35
Average	76.83	−3.73	6.32	101.3	0.10	3.43	0.201	0.035	17.54	6.79	5.86	12.64	0.89	4.87	7.95	81.83
±SD	1.01	0.66	3.68	22.55	0.02	0.10	0.042	0.024	0.28	1.35	0.53	1.62	0.18	4.61	0.81	0.46

Multivariate analysis of variance (MANOVA) in comparison of average values (*p* < 0.05). SD: standard deviation. Every average is the outcome of three determinations (*n =* 3) except those of color parameter values (L*, a*, b*) which are the outcome of five determinations (*n =* 5). TDS = total dissolved solids; EC = electrical conductivity; FA = free acidity; LA = laconic acidity; TA = total acidity; L/FA = lactonic/free acidity ratio; BI = browning index.

**Table 3:** Multivariate analysis of variance for testing the equality of the means of investigation of the physicochemical parameters according to floral type of Egyptian honeys.

Physicochemical Parameters	Wilks’ Lambda	F	df1	df2	*p*
L*	0.759	6.343	1	20	0.020
a*	0.605	13.043	1	20	0.002
b*	0.929	1.534	1	20	0.230 ^ns^
TDS	0.527	17.972	1	20	0.000
Salinity	0.537	17.255	1	20	0.000
EC	0.498	20.126	1	20	0.000
Moisture	0.706	8.320	1	20	0.009
FA	0.822	4.340	1	20	0.050
LA	0.956	0.924	1	20	0.348 ^ns^
TA	0.798	5.057	1	20	0.036
L/FA	0.903	2.159	1	20	0.157 ^ns^
BI	0.860	3.250	1	20	0.086 ^ns^
TDS/TA	0.533	17.549	1	20	0.000
Total sugars (°Bx)	0.969	0.632	1	20	0.436 ^ns^
pH	0.987	0.267	1	20	0.611 ^ns^
Ash	0.498	20.126	1	20	0.000

L*, a*, b*: colour parameters, °Bx: degrees Brix, F: Fisher’s coefficient, ns: not significant, df: degrees of freedom, *p*: probability.

**Table 4:** Standardized canonical discriminant function coefficients used in the developed statistical model for the discrimination of Egyptian honeys.

Physicochemical Parameters	Discriminant Function 1
L*	0.128
a*	0.818
TDS	−3.362
Salinity	0.221
Moisture	−0.693
FA	0.101
TA	3.041
TDS/TA	2.565

**Table 5:** Physicochemical parameter values of the ‘’unknown’’ honeys from Greece.

Botanical Origin	L*	a*	b*	pH	FA (meq/kg)	TDS (mg/L)	Salinity (g/L)	EC (mS/cm)	Ash (g/100g)	Moisture (g/100g)	Total Sugars (°Bx)	BI
Unknown honeys	68.11	−3.60	26.79	4.20	25.00	389.0	0.378	0.974	0.479	15.55	85.25	43.64
Unknown honeys	70.07	−3.80	18.30	4.90	21.50	1055.0	0.456	1.200	0.609	15.78	85.50	24.99
Unknown honeys	72.23	−4.96	21.53	4.58	20.50	976.0	0.819	1.190	0.603	16.38	82.25	28.70
Unknown honeys	68.30	−3.27	29.18	4.99	36.00	856.0	1.173	1.000	0.494	15.40	83.13	49.37
Unknown honeys	72.96	−4.03	19.11	4.23	15.73	771.0	1.048	1.885	1.003	16.32	82.30	25.01
Unknown honeys	72.96	−4.04	18.89	4.46	24.00	789.2	0.833	1.010	0.500	12.00	86.38	24.61
Average	70.77	−3.95	22.30	4.56	23.79	806.0	0.785	1.210	0.615	15.24	84.14	32.72
±SD	2.25	0.57	4.60	0.33	6.81	231.9	0.315	0.345	0.198	1.64	1.79	10.93

MANOVA in comparison of average values (*p* < 0.05). SD: standard deviation. Every average is the outcome of three determinations (*n =* 3) except those of color parameter values which are the outcome of five determinations (*n =* 5).

**Table 6:** Discriminatory power of the developed statistical model for the classification of clover and citrus honeys from Egypt against the ‘’unknown’’ honeys from Greece.

Classification Results						
		Botanical Origin	Predicted Group Membership ^c^			Total Honey Samples (N = 28)
			Clover	Citrus	Unknown	
Original ^a^	Count	Clover	15	0	0	15
		Citrus	0	7	0	7
		Unknown	0	0	6	6
	%	Clover	100.0	0	0	100.0
		Citrus	0	100.0	0	100.0
		Unknown	0	0	100.0	100.0
Cross-validated ^b^	Count	Clover	14	1	0	15
		Citrus	0	7	0	7
		Unknown	1	0	5	6
	%	Clover	93.3	6.7	0	100.0
		Citrus	0	100.0	0	100.0
		Unknown	16.7	0	83.3	100.0

^a^. 100.0% of original grouped cases correctly classified; ^b^. Cross validation was done only for those cases in the analysis. In cross validation, each case was classified by the functions derived from all cases other than that case; ^c^. 92.9% of cross-validated cases grouped cases correctly.
